# Supplementary material for: Ear, Nose and Throat (ENT) disease diagnostic error in low-resource health care: Observations from a hospital-based cross-sectional study
Source: PLoS One. 2023 Feb 9;18(2):e0281686. doi: 10.1371/journal.pone.0281686 (PMC9910637; doi:10.1371/journal.pone.0281686)
Supplement: S1 Appendix — (DOCX) [file pone.0281686.s001.docx]

| Date | File # of patient | Name of patient | Sex | Age | Town of residence | Province of residence | Contact phone number | Place of referral | Province of referral | UTH referral or not? | UTH Hospital/dept | Referral facility level of care | Referral diagnosis 1 | Reason for referral | Diagnosis match | ENT diagnosis 1 | Additional ENT diagnosis information | Specialty ENT diagnosis 1 | Attendance (maiden, review) | referral appropriateness | Reason for referral appropriateness |
| --- | --- | --- | --- | --- | --- | --- | --- | --- | --- | --- | --- | --- | --- | --- | --- | --- | --- | --- | --- | --- | --- |
|  |  |  |  |  |  |  |  |  |  |  |  |  |  |  |  |  |  |  |  |  |  |

S1 Appendix: Data collecting tool

*ENT: Ear, Nose and Throat; UTH: University Teaching Hospital*
