# Supplementary material for: Ear, Nose and Throat (ENT) disease diagnostic error in low-resource health care: Observations from a hospital-based cross-sectional study
Source: PLoS One. 2023 Feb 9;18(2):e0281686. doi: 10.1371/journal.pone.0281686 (PMC9910637; doi:10.1371/journal.pone.0281686)
Supplement: S2 Appendix — (DOCX) [file pone.0281686.s002.docx]

S2 Appendix: The STROBE checklist for cross sectional studies

|  | **Item No** | **Page Number** | **Recommendation** |
| --- | --- | --- | --- |
| **Title and abstract** | 1 | 1 | (*a*) Indicate the study’s design with a commonly used term in the title or the abstract |
|  |  | 2 | (*b*) Provide in the abstract an informative and balanced summary of what was done and what was found |
| **Introduction** | | | |
| Background/rationale | 2 | 2-3 | Explain the scientific background and rationale for the investigation being reported |
| Objectives | 3 | 3 | State specific objectives, including any prespecified hypotheses |
| **Methods** | | | |
| Study design | 4 | 3 | Present key elements of study design early in the paper |
| Setting | 5 | 3-6 | Describe the setting, locations, and relevant dates, including periods of recruitment, exposure, follow-up, and data collection |
| Participants | 6 | 4-5 | (*a*) Give the eligibility criteria, and the sources and methods of selection of participants |
| Variables | 7 | 4-6 | Clearly define all outcomes, exposures, predictors, potential confounders, and effect modifiers. Give diagnostic criteria, if applicable |
| Data sources/ measurement | 8 | 4-6 | For each variable of interest, give sources of data and details of methods of assessment (measurement). Describe comparability of assessment methods if there is more than one group |
| Bias | 9 | 16-17 | Describe any efforts to address potential sources of bias |
| Study size | 10 | 3-4 | Explain how the study size was arrived at |
| Quantitative variables | 11 | 4-6 | Explain how quantitative variables were handled in the analyses. If applicable, describe which groupings were chosen and why |
| Statistical methods | 12 | 5-6 | (*a*) Describe all statistical methods, including those used to control for confounding |
|  |  | 5-6 | (*b*) Describe any methods used to examine subgroups and interactions |
|  |  | 4-6 | (*c*) Explain how missing data were addressed |
|  |  | 5-6 | (*d*) If applicable, describe analytical methods taking account of sampling strategy |
|  |  | Not applicable | (*e*) Describe any sensitivity analyses |
|  | | **Results** | |
| Participants | 13 | 6-10 | (a) Report numbers of individuals at each stage of study—eg numbers potentially eligible, examined for eligibility, confirmed eligible, included in the study, completing follow-up, and analyzed |
|  |  | 6-10 | (b) Give reasons for non-participation at each stage |
|  |  | Narrative used | (c) Consider use of a flow diagram |
| Descriptive data | 14 | 6-10 | (a) Give characteristics of study participants (eg demographic, clinical, social) and information on exposures and potential confounders |
|  |  | 6-10 | (b) Indicate number of participants with missing data for each variable of interest |
| Outcome data | 15 | 6-10 | Report numbers of outcome events or summary measures |
| Main results | 16 | 6-10 | (*a*) Give unadjusted estimates and, if applicable, confounder-adjusted estimates and their precision (eg, 95% confidence interval). Make clear which confounders were adjusted for and why they were included |
|  |  | 6-10 | (*b*) Report category boundaries when continuous variables were categorized |
|  |  | Not relevant | (*c*) If relevant, consider translating estimates of relative risk into absolute risk for a meaningful time period |
| Other analyses | 17 | 6-10 | Report other analyses done—eg analyses of subgroups and interactions, and sensitivity analyses |
|  | | **Discussion** | |
| Key results | 18 | 11-16 | Summarise key results with reference to study objectives |
| Limitations | 19 | 16-17 | Discuss limitations of the study, taking into account sources of potential bias or imprecision. Discuss both direction and magnitude of any potential bias |
| Interpretation | 20 | 11-16 | Give a cautious overall interpretation of results considering objectives, limitations, multiplicity of analyses, results from similar studies, and other relevant evidence |
| Generalisability | 21 | 11-16 | Discuss the generalisability (external validity) of the study results |
|  | | **Other information** | |
| Funding | 22 | Included on the submission form | Give the source of funding and the role of the funders for the present study and, if applicable, for the original study on which the present article is based |
