# Supplementary material for: Ear, Nose and Throat (ENT) disease diagnostic error in low-resource health care: Observations from a hospital-based cross-sectional study
Source: PLoS One. 2023 Feb 9;18(2):e0281686. doi: 10.1371/journal.pone.0281686 (PMC9910637; doi:10.1371/journal.pone.0281686)
Supplement: S1 Table — (DOCX) [file pone.0281686.s003.docx]

S1 Table: Proportional representation of ENT diseases treated at UTH ENT clinic

| Variable | Frequency, number n (%) | | | | | |
| --- | --- | --- | --- | --- | --- | --- |
|  | Head and Neck diseases,  n (%) | Ear diseases,  n (%) | Sinonasal diseases,  n (%) | Medical problem,  n (%) | No ENT pathology, n (%) | p-value |
| ***Age group of patient (years)*** |  | | | | | |
| 0-5 | 152 (37.5) | 49 (12.1) | 192 (47.4) | 4 (1.0) | 8 (2.0) | 0.000* |
| 6-12 | 116 (48.7) | 24 (10.1) | 86 (36.1) | 1 (0.4) | 11 (4.6) |  |
| 13-18 | 32 (37.7) | 25 (29.4) | 25 (29.4) | 0 | 3 (3.5) |  |
| 19-35 | 118 (33.3) | 123 (34.8) | 93 (26.3) | 1 (0.3) | 19 (5.4) |  |
| 36-50 | 69 (29.0) | 87 (36.6) | 67 (28.2) | 1 (0.4) | 14 (5.9) |  |
| 51-87 | 50 (22.9) | 89 (40.8) | 67 (30.7) | 2 (0.9) | 10 (4.6) |  |
| ***Gender, n*** |  | | | | | |
| Male | 237 (32.8) | 195 (27.0) | 258 (35.9) | 0 | 31 (4.3) | 0.025* |
| Female | 300 (36.8) | 202 (24.8) | 271 (32.2) | 9 (1.1) | 34 (4.2) |  |
| ***Province of referral*** |  | | | | | |
| Central | 6 (42.9) | 4 (28.6) | 4 (28.6) | 0 | 0 | 0.997* |
| Copperbelt | 14 (38.9) | 7 (19.4) | 13 (36.1) | 0 | 2 (5.6) |  |
| Eastern | 6 (60.0) | 2 (20.0) | 2 (20.0) | 0 | 0 |  |
| Luapula | 2 (33.3) | 2 (33.3) | 2 (33.3) | 0 | 0 |  |
| Lusaka | 442 (34.7) | 327 (25.7) | 441 (34.6) | 8 (0.6) | 56 (4.4) |  |
| Muchinga | 3 (75.0) | 0 | 1 (25.0) | 0 | 0 |  |
| Northern | 2 (50.0) | 0 | 2 (50.0) | 0 | 0 |  |
| Southern | 7 (38.9) | 2 (11.1) | 8 (44.4) | 0 | 1 (5.6) |  |
| Western | 2 (66.7) | 0 | 1 (33.3) | 0 | 0 |  |
| ***Referring facility level of care*** |  | | | | | |
| Clinic/health centre | 33 (39.3) | 18 (21.4) | 30 (35.7) | 0 | 3 (3.6) | 0.745* |
| Level 1 | 47 (42.7) | 21 (19.1) | 36 (32.7) | 0 | 6 (5.5) |  |
| Level 2 | 22 (40.7) | 11 (20.4) | 19 (35.2) | 0 | 2 (3.7) |  |
| Level 3 | 382 (34.1) | 294 (26.3) | 388 (34.6) | 8 (0.7) | 48 (4.3) |  |

**Note: some cells have frequencies less than or equal to 5; thus, Fisher's test was applied.*
