# Supplementary material for: Ear, Nose and Throat (ENT) disease diagnostic error in low-resource health care: Observations from a hospital-based cross-sectional study
Source: PLoS One. 2023 Feb 9;18(2):e0281686. doi: 10.1371/journal.pone.0281686 (PMC9910637; doi:10.1371/journal.pone.0281686)
Supplement: S2 Table — (DOCX) [file pone.0281686.s004.docx]

S2 Table: Misdiagnosis across age groups, ENT subspecialties, referral facilities level of care and provinces

| Variable | Frequency, number n (%) | | | | | | | | | | | | | | | | |
| --- | --- | --- | --- | --- | --- | --- | --- | --- | --- | --- | --- | --- | --- | --- | --- | --- | --- |
| **Diagnosis match** | 1. **Age range (years)** | | | | | | | | | | | | | | | | p-value |
|  | ***0-5 yrs*** | | ***6-12 yrs*** | | | | ***13-18 yrs*** | | | ***19-35yrs*** | | ***36-50 yrs*** | | | ***51-78 yrs*** | | 0.000 |
| No | 230 (62.5) | | 130 (61.9) | | | | 40 (54.8) | | | 219 (69.1) | | 152 (74.9) | | | 145 (77.1) | |  |
| Yes | 138 (37.5) | | 80 (38.1) | | | | 33 (45.2) | | | 98 (30.9) | | 51 (25.1) | | | 43 (22.9) | |  |
|  | 1. **Province of referral** | | | | | | | | | | | | | | | | |
|  | East | Cent | | CB | | | Luap | | LSK | | Much | North | Sout | | | West | 0.784* |
| No | 6 (60.0) | 8(57.1) | | 24 (66.7) | | | 5 (83.3) | | 850(67.6) | | 3 (75.0) | 2 (50.0) | 14(77.8) | | | 1 (33.3) |  |
| Yes | 4 (40.0) | 6(42.9) | | 12 (33.3) | | | 1 (16.7) | | 410 (32.5) | | 1 (25.0) | 2 (50.0) | 4 (22.2) | | | 2 (66.7) |  |
|  | 1. **Referral facility level of care** | | | | | | | | | | | | | | | | |
|  | ***Clinic/Health Centre*** | | | | ***Level 1*** | | | | | ***Level 2*** | | | | ***Level 3*** | | | 0.495 |
| No | 56 (66.7) | | | | 80 (74.1) | | | | | 36 (66.7) | | | | 741 (66.8) | | |  |
| Yes | 28 (33.3) | | | | 28 (25.9) | | | | | 18 (33.3) | | | | 368 (33.2) | | |  |
|  | 1. **ENT subspecialty** | | | | | | | | | | | | | | | | |
|  | ***Head and Neck*** | | ***Otology*** | | | | | ***Rhinology*** | | | ***Medical problem*** | | ***No ENT pathology*** | | | | 0.000* |
| No | 282 (58.3) | | 239 (71.6) | | | | | 333 (70.3) | | | 3 (42.9) | | 55 (98.2) | | | |  |
| Yes | 202 (41.7) | | 95 (28.4) | | | | | 141 (29.7) | | | 4 (57.1) | | 1 (1.8) | | | |  |
|  | 1. **Referring department/Hospital within UTH** | | | | | | | | | | | | | | | | |
|  | ***Medicine*** | | | | | ***Paediatrics*** | | | | | ***Surgery*** | | ***Women and Newborn*** | | | | 0.000* |
| No | 151 (77.8) | | | | | 169 (57.1) | | | | | 397 (68.0) | | 1 (33.3) | | | |  |
| Yes | 43 (22.2) | | | | | 127 (42.9) | | | | | 187 (32.0) | | 2 (66.7) | | | |  |

**Note: some cells have frequencies less than or equal to 5; thus, Fisher's test was applied*

*Province abbreviations: East, Eastern; Cent, Central; CB, Copperbelt; Luap, Luapula; LSK, Lusaka; Much, Muchinga; North, Northern; South, Southern; West, Western; N-West, North-Western*
