# Supplementary material for: Ear, Nose and Throat (ENT) disease diagnostic error in low-resource health care: Observations from a hospital-based cross-sectional study
Source: PLoS One. 2023 Feb 9;18(2):e0281686. doi: 10.1371/journal.pone.0281686 (PMC9910637; doi:10.1371/journal.pone.0281686)
Supplement: S3 Table — (DOCX) [file pone.0281686.s005.docx]

S3 Table: Appropriateness of referral across age groups, ENT subspecialties, referral facilities level of care and provinces

| Variable | Frequency, number n (%) | | | | | | | | | | | | | | | | | |
| --- | --- | --- | --- | --- | --- | --- | --- | --- | --- | --- | --- | --- | --- | --- | --- | --- | --- | --- |
| **Appropriate referral** | 1. **Age range (years)** | | | | | | | | | | | | | | | | | p-value |
|  | ***0-5 yrs*** | | ***6-12 yrs*** | | | | ***13-18 yrs*** | | | ***19-35yrs*** | | ***36-50 yrs*** | | | ***51-78 yrs*** | | | 0.058 |
| **No** | 213 (57.1) | | 120 (55.3) | | | | 36 (49.3) | | | 202 (63.3) | | 111 (54.1) | | | 95 (50.8) | | |  |
| **Yes** | 160 (42.9) | | 97 (44.7) | | | | 37 (50.7) | | | 117 (36.7) | | 94 (45.9) | | | 92 (49.2) | | |  |
|  | 1. **Province of referral** | | | | | | | | | | | | | | | | | |
|  | East | Cent | | CB | | | Luap | LSK | | Much | North | | Sout | | | West | | 0.021* |
| **No** | 3 (33.3) | 5 (35.7) | | 13 (36.1) | | | 3 (50.0) | 741(58.0) | | 2 (50.0) | 1(25.0) | | 7 (38.9) | | | 1 (33.3) | |  |
| **Yes** | 6 (66.7) | 9 (64.2) | | 23 (63.9) | | | 3 (50.0) | 536(42.0) | | 2 (50.0) | 3(75.0) | | 11(61.1) | | | 2 (66.7) | |  |
|  | 1. **Referral facility level of care** | | | | | | | | | | | | | | | | | |
|  | ***Clinic/Health Centre*** | | | | | ***Level 1*** | | | | ***Level 2*** | | | | ***Level 3*** | | | 0.044 | |
| **No** | 50 (59.5) | | | | | 62 (56.4) | | | | 20 (37.7) | | | | 642 (57.2) | | |  |  |
| **Yes** | 34 (40.5) | | | | | 48 (43.6) | | | | 33 (62.3) | | | | 481 (42.8) | | |  |  |
|  | 1. **ENT subspecialty** | | | | | | | | | | | | | | | | | |
|  | ***Head and Neck*** | | ***Otology*** | | | | ***Rhinology*** | | | ***Medical problem*** | | | ***No ENT pathology*** | | | | 0.000 | |
| **No** | 302 (62.4) | | 156 (45.3) | | | | 258 (54.3) | | | 3 (37.5) | | | 54 (91.5) | | | |  |  |
| **Yes** | 182 (37.6) | | 188 (54.7) | | | | 217 (45.7) | | | 5 (62.5) | | | 5 (8.5) | | | |  |  |
|  | 1. **Referring department/Hospital within UTH** | | | | | | | | | | | | | | | | | |
|  | ***Medicine*** | | | | ***Paediatrics*** | | | | ***Surgery*** | | | ***Women and Newborn*** | | | | | 0.821* | |
| **No** | 114 (57.6) | | | | 183 (60.2) | | | | 335 (57.1) | | | 2 (66.7) | | | | |  |  |
| **Yes** | 84 (42.4) | | | | 121 (39.8) | | | | 252 (42.9) | | | 1 (33.3) | | | | |  |  |

**Note: some cells have frequencies less than or equal to* *5; thus, Fisher's test was applied*

*Province abbreviations: East, Eastern; Cent, Central; CB, Copperbelt; Luap, Luapula; LSK, Lusaka; Much, Muchinga; North, Northern; South, Southern; West, Western; N-West, North-Western.*
